# Differential Mechanisms of Cell Death Induced by HDAC Inhibitor SAHA and MDM2 Inhibitor RG7388 in MCF-7 Cells

**DOI:** 10.3390/cells8010008

**Published:** 2018-12-22

**Authors:** Umamaheswari Natarajan, Thiagarajan Venkatesan, Vijayaraghavan Radhakrishnan, Shila Samuel, Appu Rathinavelu

**Affiliations:** 1 Rumbaugh-Goodwin Institute for Cancer Research, Nova Southeastern University, Ft. Lauderdale, FL 33314, USA; tvenkatesan@nova.edu; 2 VRR Institute of Biomedical Sciences, Kattupakkam, Chennai 600056, India; ubaruthrarini@gmail.com or un15@mynsu.nova.edu (U.N.); drrvijayaraghavan@gmail.com (V.R.); vrribms@gmail.com (S.S.); 3 College of Pharmacy, Health Professions Division, Nova Southeastern University, Ft. Lauderdale, FL 33314, USA

**Keywords:** SAHA, RG7388, necroptosis, apoptosis, p21, phospho-RIP3, MLKL, MCF-7

## Abstract

Gene expression is often altered by epigenetic modifications that can significantly influence the growth ability and progression of cancers. SAHA (Suberoylanilide hydroxamic acid, also known as Vorinostat), a well-known Histone deacetylase (HDAC) inhibitor, can stop cancer growth and metastatic processes through epigenetic alterations. On the other hand, Letrozole is an aromatase inhibitor that can elicit strong anti-cancer effects on breast cancer through direct and indirect mechanisms. A newly developed inhibitor, RG7388 specific for an oncogene-derived protein called MDM2, is in clinical trials for the treatment of various cancers. In this paper, we performed assays to measure the effects of cell cycle arrest resulting from individual drug treatments or combination treatments with SAHA + letrozole and SAHA + RG7388, using the MCF-7 breast cancer cells. When SAHA was used individually, or in combination treatments with RG7388, a significant increase in the cytotoxic effect was obtained. Induction of cell cycle arrest by SAHA in cancer cells was evidenced by elevated p21 protein levels. In addition, SAHA treatment in MCF-7 cells showed significant up-regulation in phospho-RIP3 and MLKL levels. Our results confirmed that cell death caused by SAHA treatment was primarily through the induction of necroptosis. On the other hand, the RG7388 treatment was able to induce apoptosis by elevating BAX levels. It appears that, during combination treatments, with SAHA and RG7388, two parallel pathways might be induced simultaneously, that could lead to increased cancer cell death. SAHA appears to induce cell necroptosis in a p21-dependent manner, and RG7388 seems to induce apoptosis in a p21-independent manner, outlining differential mechanisms of cell death induction. However, further studies are needed to fully understand the intracellular mechanisms that are triggered by these two anti-cancer agents.

## 1. Introduction

Breast cancer is one of the most common causes of cancer-related deaths worldwide. Despite continued efforts around the globe, there have been only marginal improvements in breast cancer related treatment success. The median survival time for patients with metastatic breast cancer is no more than one year [1]. Therefore, the need for new efficient drugs, and treatment strategies for effective treatment of breast cancer remains. Breast cancer development is triggered by the complex process of tumorigenesis, it is also intimately linked to gene mutation and abnormal gene expression. In fact, epigenetic changes are also very common and significant in the tumorigenesis process of breast cancers, as these changes affect gene transcription without modifying the underlying DNA sequence, and can steadily lead to continuous cell division [2,3]. In normal breast cells, histones are modified via acetylation, methylation, and phosphorylation to meet certain cell function needs [2,3]. Acetylation of histones is one of the most common epigenetic modifications regulated largely by histone acetyltransferases (HATs), which transfer the hydrophobic acetyl group from acetyl coenzyme A (Acetyl CoA) to specific lysine residues on the N-terminal tails of histones H2A, H2B, H3, and H4 [4]. This addition leads to neutralization of the positive charge on amino groups and increases the steric interference. This results in loosening of the histone-DNA structure that is more accessible for transcription machinery and gene activation. On the other hand, DNA stretching and activation of gene expression can be turned off when histones are deacetylated [5,6]. Histone deacetylation is carried out by enzymes that belong to two families; the classical HDAC and Silent information regulator 2 (SIR2) families [7]. There are 11 HDAC isoenzymes that deacetylate histones within the nucleus, and specific HDACs are differentially regulated to modulate the expression of various groups of genes [8]. Classical HDACs can be split into three classes (I, II, and IV) based on phylogeny. Class I contains 4 members (HDACs 1, 2, 3, and 8) each containing a deacetylase domain, which exhibits from 45% to 93% similarity in amino acid sequence related to the yeast Rpd3 (reduced potassium dependency-3) protein [9,10]. Class II HDACs (4, 5, 6, 7, 9, and 10) are most closely related to yeast Hda1, and are found in both the nucleus and cytoplasm. Class III HDACs are related to the yeast Sirt2 gene, and form a structurally discrete class of NAD-dependent deacetylase enzymes that are also found in the nucleus as well as in the cytoplasm [11]. Gene expression can be activated when histone deacetylase inhibitors are used, which induces growth arrest, autophagy, necroptosis, apoptosis, anti-angiogenesis, and suppression of the cell or tissue differentiation. This indicates that the net effect of histone acetylation blockade is an inhibition of cell proliferation. Histone deacetylase inhibitors (HDACi) have been reported to stimulate much enthusiasm in the field of oncology with more than 34 clinical trials initiated to date. These have resulted in clinical approval by the Food & Drug Administration (FDA) of SAHA—Suberoylanilide hydroxamic acid (also known as vorinostat)—and few other antitumor agents with HDACi (Histone Deacetylase Inhibitor) ability. HDACi’s are used to treat continuously deteriorating or recurrent T-cell lymphomas and breast cancer [12]. HDACi’s effectively inhibit HDAC activity, and increase histone acetylation to commensurate cancer cells with the necessary transcription to induce cell differentiation, arrest of cell cycle progression, and induction of cancer cell death. Several studies have so far confirmed that SAHA can inhibit the expression of cyclin D1 and activate p21 (WAF1/CIP1) function, resulting in cancer cell cycle arrest and induction of cancer cell death [13,14]. The function of HDACi’s are not only limited to mediating acetylation of histones, but also involved with the modification of several non-histone proteins [15,16]. In the treatment of breast cancer, aromatase inhibitors (AIs) also play an important role. They block the aromatase enzyme and prevent the conversion of androstenedione to estrone, which may play a significant role in tumor proliferation if the cancer cells are hormone dependent [17,18]. Letrozole is currently used in neo-adjuvant, adjuvant, and extended adjuvant chemotherapies in post-menopausal, ERα-positive patient’s metastatic disease [19]. Therefore, the use of letrozole in combination with standard breast cancer treatment drugs holds remarkable clinical potential. 

The tumor suppressor gene p53 plays a vital role in transcription and cellular regulation processes [20,21,22,23,24,25]. Among several mechanisms that can interfere with the cell cycle regulatory function of p53, the MDM2-mediated inhibition is one of the most significant, which can produce cellular and mechanistic consequences similar to p53 mutation [26,27,28]. However, the well-defined interface of MDM2–p53 binding has made it possible to design small-molecule inhibitors to target MDM2 and restore cell cycle regulation. The latest generation of MDM2 inhibitors, including RG7388, has demonstrated selective and potent MDM2 inhibition with improved bioavailability [29]. Several studies conducted by utilizing RG7388, to effectively rescue p53 and activate downstream apoptotic pathways in p53 wild-type cell lines including breast cancer cells, have yielded positive results [30]. MDM2 inhibitors have also been found to produce therapeutic effects through p53 independent mechanisms. Therefore, we were interested in exploring the potential use of HDAC and MDM2 inhibitors in combination, to provide cell cycle arrest and related therapeutic benefits. The inhibition of HDAC and MDM2 have been reported to increase the expression of p21 through different mechanisms, eventually leading to cell cycle arrest and cell death. We therefore wanted to determine any possible synergy or interplay between the mechanisms triggered by these two drugs. Cell death is caused by drug treatment complexes, and can be mediated through a wide range of mechanisms [31], among which necroptosis is defined as the caspase independent, regulated cell death (RCD). During necroptosis, RIP3 has been confirmed as an indispensable protein, which is reported to execute the cell death process with help from the mixed lineage kinase domain-like (MLKL) protein. Some of the earlier studies have shown that during TNF-induced necroptosis, ROS production is RIP3-dependent, which in turn can activate the metabolic enzymes glycogen phosphorylase L (PYGL), glutamate-ammonia ligase (GLUL), and glutamate dehydrogenase 1 (GLUD1), leading to enhanced aerobic respiration and TNF-induced ROS production [32]. In addition, HtrA2/Omi and UCH-L1 have been suggested as two crucial proteases involved in TNF-induced necroptosis, and their role is supported by strong evidence that proteolysis is not only critical for the regulation and execution of apoptosis, but also essential for caspase-independent forms of RCD [33]. In addition, olaparib—a selective PARP-1 inhibitor—failed to block TNF-induced necroptosis in L929 cells. This was identical to the knockdown and knockout of PARP-1, indicating that necroptosis can be executed without PARP-1 [34]. In the current study, we evaluated the effectiveness of SAHA, letrozole, and RG7388 on cell cycle arrest and RCD following individual and combination treatments.

## 2. Materials and Methods

### 2.1. Reagents and Antibodies

The histone deacetylase (HDAC) inhibitor SAHA was purchased from Selleckchem (Houston, TX, USA), the MDM2 inhibitor RG7388 was purchased from MedChemExpress (Monmouth Junction, NJ, USA). Letrozole was purchased from Selleckchem (Houston, TX, USA), and Necrostatin was purchased from Abcam (Cambridge, MA, USA). The primary antibodies against p53, phsoph-p53, p21, CDC25C, TIPM-1, CDK1, BAK, BAX, APAF1, Bcl-XL, RIP1, and cleaved PARP (1:1000) were purchased from Cell Signaling Technology (Danvers, MA, USA). Phospho-RIP3 and MLKL (1:1000) antibodies were purchase from Abcam (Cambridge). MDM2 (1:500) was purchased from Santa Cruz biotechnology (Dallas, TX, USA). The β-actin antibody (1:2000) was purchased from Sigma Aldrich chemical company (St. Louis, MO, USA). The secondary antibodies anti-rabbit, anti-mouse, HRP conjugated, and DMSO were purchased from Sigma Aldrich chemical company. Nitrocellulose membranes (0.45 µm) were purchased from Amersham (GE Healthcare Life Sciences, Marlborough, MA, USA). ECL was purchased from KPL biosolutions (Milford, MA, USA). SYTOX^®^ Green and DEVD-*amc* CellEvent^TM^ Caspase-3/7 Green ReadyProbe^TM^ were purchased from Thermo Fisher (Molecular Probes, Life Technologies, Carlsbad, CA, USA). All other chemicals used in this study were of research grade.

### 2.2. Cell Culture and Drug Treatments

Human breast adenocarcinoma cell line (MCF-7) was obtained from the American Type Culture Collection (Manassas, VA, USA). MCF-7 cells were cultured with Dulbecco’s Modified Eagle’s Medium (DMEM), supplemented with 10% fetal bovine serum (FBS), 1% Amphotericin B and 1% Penicillin G- treptomycin. The cells were maintained in a humidified atmosphere with 95% air and 5% CO_2_ at 37 °C. When MCF-7 cells reached 75–80% confluence, they were treated with 7.5 µM SAHA, 100 nM letrozole, 2.0 µM RG7388, combination of SAHA + letrozole (7.5 µM + 100 nM), or SAHA + RG7388 (7.5 µM + 2.0 µM) for 24 h. After incubation the cells were used for protein extraction, and western blot analysis. Similarly, the cell viability assays, flow cytometry analysis, and fluorescence staining were performed after cell treatment with the above-mentioned scheme. Untreated MCF-7 cells were used as control for all our comparative analyses, and all experiments were performed in triplicates.

### 2.3. Cell Viability Assessment Using Trypan Blue Dye Exclusion Method 

MCF-7 cells were plated at a density of 5 × 10^3^ cells/well in 96-well plates, and incubated at 37 °C under 95% air and 5% CO_2_ for 24 h. The experiments were performed with cells that have gone through no more than 10 passages from the time they were revived from our master bank stored in liquid nitrogen. Once the cells reached 75–80% confluence, they were treated with different concentrations of the drugs, in individual or combination treatments for 24 h. After incubation, cell viability was assessed using Trypan blue dye exclusion method (TBDE). After removing the incubation medium, equal amounts of 0.4% trypan blue dye and the cell suspension were added. The mixture was allowed to incubate for less than three minutes at room temperature. Cell viability was counted using the TC20 automated cell counter from Bio-Rad (Hercules, CA, USA).

### 2.4. Protein Preparation and Western Blot Analysis 

After 24 h of treatment, cells were lysed with RIPA (Radio-Immunoprecipitation Assay) buffer, containing the protease inhibitor cocktail and sodium orthovanadate (Santa Cruz Inc., Dallas, TX, USA), for 30 min at 4 °C. Cell lysates were clarified by centrifugation at 4 °C for 20 min at 14,000 rpm, and then protein concentrations were determined using the bicinchoninic acid (BCA) protein assay method (Thermo Fisher Scientific, Grand Island, NY, USA). For western blot analysis, equal concentrations of protein were separated using sodium dodecyl sulfate-polyacrylamide (7.5%, 10%, and 12% were used as per the molecular weight of the proteins) gel electrophoresis (SDS-PAGE), and blotted onto a nitrocellulose membrane. After protein transfer, the membranes were blocked using the proteins from non-fat dry milk and probed with specific antibodies for MDM2, p53, phospho-p53, p21, CDC25C, TIMP-1, CDK1, BAX, BAK, APAF1, Bcl-XL, RIP1, phospho-RIP3, MLKL, cleaved PARP, and β-actin. Finally, for the detection of specific proteins, membranes were incubated in a solution containing the LumiGLO Reserve Chemiluminescent substrate. Densitometric analyses were performed using the ImageJ program.

### 2.5. Flow Cytometry Analysis 

When the MCF-7 cells reached 75–80% confluence, they were harvested and transferred with complete medium into 1.5 ml reaction tubes. Cells were centrifuged at 500× *g* for 5 min at 4 °C, the supernatant was removed, and after washing them with 1 ml PBS, the cells were resuspended in 100 μL staining buffer (Annexin V-FITC staining Kit, Roche) by mixing 0.2 μL of Annexin-V-FITC and 2 μL propidium iodide (PI) in incubation buffer according to manufacturer’s instructions. The cells were incubated for 15 min at room temperature. Finally, cells were diluted to 500 μL with PBS and analyzed immediately with the Accuri 6 flow cytometer (BD Biosciences, San Jose, CA, USA). During analysis, the 488 nm laser was used for excitation. Debris and doublets were gated out. Bright field (430–480 nm), Annexin V-FITC (505–560 nm), and PI (595–642 nm) channels were measured and at least 5000 events of single cells per sample were collected. Color compensation was necessary as FITC and PI have overlapping emission spectra. Additional single-labeled samples were prepared, which contained dead cells and served as a positive control for single staining of Annexin V-FITC or PI. For analysis, the IDEAS version 6.0 was used and the Gating strategy was the following: Depending on fluorescence intensity of Annexin V-FITC and PI, the populations were distinguished into double negative (healthy), Annexin-V positive (early apoptotic cells), and double positive (late apoptotic or necroptotic) cells.

### 2.6. Fluorescence Imaging and Assay for Cell Death Assessment

SYTOX^®^ Green is a high-affinity nucleic acid stain that can easily penetrate cells when the plasma membrane integrity is compromised. However, SYTOX Green does not cross the plasma membranes of live cells. Therefore, when drug treated cells are incubated with SYTOX Green, the nucleic acids of dead cells bind the dye and emit bright green fluorescence when excited at 503 nm. Since the level of fluorescence increases >500-fold upon nucleic acid binding, SYTOX Green stain is a simple and quantitative single-step dead-cell indicator method for use with fluorescence microscopes and fluorescence spectrophotometer analyses [35]. The fluorescent caspase substrate DEVD-*amc* is also cell-impermeable. The caspase substrate DEVD-*amc* remains unprocessed and is nonfluorescent. Apoptotic cells typically proceed to a stage of secondary necrosis in vitro, resulting in SYTOX Green entry, nuclear DNA binding and an increase in fluorescence intensity. However, apoptotic cells contain active caspases, and therefore can cleave DEVD-*amc*, which is a fluorogenic substrate for caspase-3. The peptide sequence is based on the PARP cleavage site Asp^216^ for caspase-3. Once cleaved from DEVD, the *amc* (7-Amino-4-methylcoumarin) can be excited between 485 nm to emit fluorescence that is measured at 535 nm. DEVD-*amc* cleavage is used to detect apoptosis using 96-well plates, by mixing 50 µL of cell lysis supernatant with 50 µM of 2× reaction mix (10 mM PIPES (pH 7.4), 2 mM EDTA, 0.1% CHAPS, and 10 mM DTT) containing 200 nM of the fluorogenic substrate Acetyl-Asp-Glu-Val-Asp-7-Amino-4- methylcoumarin (DEVD-*amc*; Enzo Life Sciences). Fluorescence was quantified using a microplate reader (excitation/emission 485/530 nm) at the start of the reaction and after 30 min. To determine the effects of the drugs, cells were treated individually with SAHA, letrozole, RG7388, and the combinations SAHA + letrozole, or SAHA + RG7388 for 24 h. After the incubation, cells were washed and incubated with the SYTOX green and DEVD-*amc* substrates. Fluorescence was measured using the Victor 3 Spectrofluorometer (Perkin Elmer, Waltham, MA, USA).

### 2.7. Cell Migration Assay

The effects of SAHA and RG7388 on cell migration were assessed using the scratch assay. A confluent monolayer of MCF-7 cells was grown on 24-well plates. A sterile 200 μL tip was used to scratch a straight line, and fresh medium with different concentrations of SAHA (0.5–10.0 μM) and RG7388 (1.0–5.0 μM) were added as single agents or in combination to the scratched monolayer. Cells in medium without any drugs were considered as control. Images were captured using a Leica microscope (DMI3000 B) at 0, 12, 24, and 36 h post-scratch. Markings were created and used as reference points close to the scratch to obtain the same field during image acquisition.

### 2.8. Statistical Analysis

The data presented are the mean ± SD from statistical significance between the groups. The data was analyzed by one-way analysis of variance (ANOVA) followed by LSD (Least Significant Difference) test. *P* < 0.05 was considered statistically significant.

## 3. Results

### 3.1. Reduction of MCF-7 Cell Viability by SAHA, Letrozole, and RG7388

**Figure 1:**
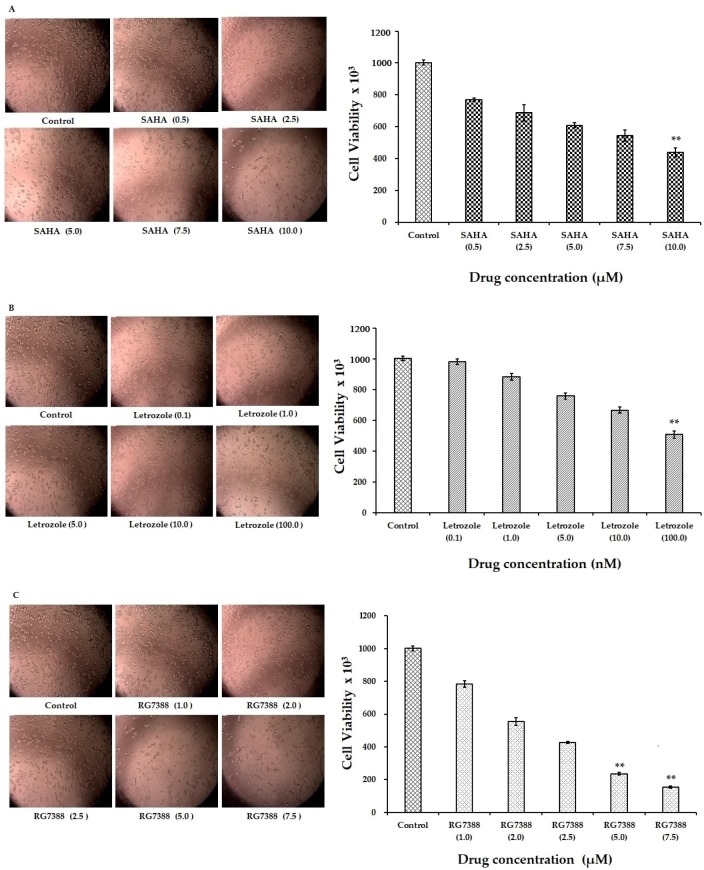

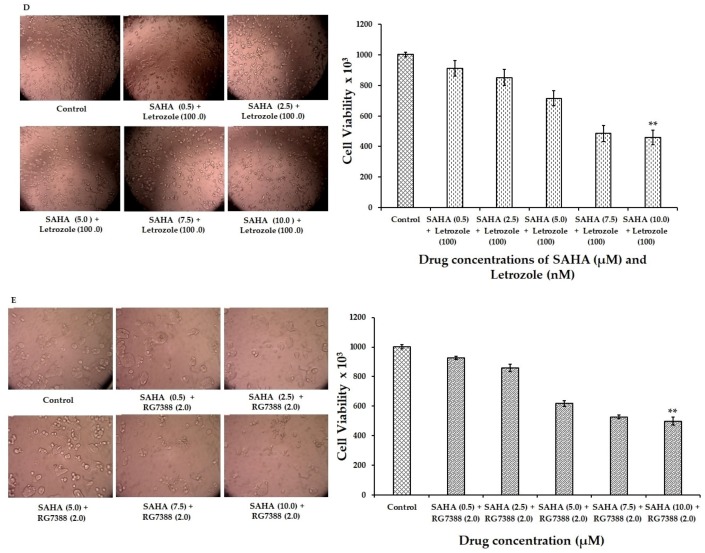
Representative images of MCF-7 cells with different concentrations of anticancer drug treatments. (**A**) The effects of 0.5, 2.5, 5.0, 7.5, and 10.0 µM of SAHA treatment on MCF-7 cells. (**B**) Illustration of the effects of letrozole (0.1, 1.0, 5.0, 10.0, and 100 nM) treatment on MCF-7 cells. (**C**) Images showing effects of RG7388 (1.0, 2.0, 2.5, 5.0 and 7.5 µM) treatment on MCF-7 cells. (**D**,**E**) MCF-7 cells treated with SAHA + letrozole and SAHA + RG7388 combinations. Analysis of cell viability after anticancer drug treatments by TBDE are shown in the corresponding bar graphs. ** *p* < 0.01.

The cytotoxic effects of SAHA, letrozole, and RG7388 on MCF-7 cells were monitored using the Trypan Blue Dye Exclusion (TBDE) method. The results of the cell viability without cell staining are shown in Figure 1A–E. The dose response effects of SAHA, letrozole, and RG7388 following individual treatments are shown in Figure 1A–C, respectively. The effects of SAHA + letrozole, and SAHA + RG7388 are shown in Figure 1D,E. While the maximum concentration tested for each drug was able to produce significant cell death, the combination of SAHA + RG7388 was more effective compared to the SAHA + letrozole. In addition, the cell count results obtained using the TBDE method are shown in Figure 1A–E. This data further confirmed that cell viability was reduced significantly by SAHA in a concentration dependent manner after the 24 h treatment. As anticipated, treatment with letrozole and RG7388 also showed reduction in the cell viability. In addition, the combination of SAHA + letrozole and SAHA + RG7388 caused significant decreases in cell viability. In treatments with SAHA (10.0 μM) and letrozole (100 nM), nearly 60% of the cells were killed after the 24 h treatment, whereas RG7388 (7.5 µM) treatment was able to kill nearly 70% of cells. Similarly, the combination treatments with SAHA + letrozole and SAHA + RG7388 produced nearly 80% cell death. From our cell viability results, approximate IC50 values of SAHA, letrozole, and RG7388 were calculated as 7.5 µM, 100 nM, and 2.0 µM, respectively in MCF-7 cells after 24 h treatment. Once the IC50 values were determined, we selected 7.5 µM, 100 nM, and 2.0 µM concentrations for SAHA, letrozole, and RG7388 for further treatments in order to maximize the effects, and to subsequently identify the intracellular mechanisms of action of the above listed drugs.

### 3.2. Induction of Apoptosis/Necroptosis in MCF-7 Cells Following SAHA, Letrozole and RG7388 Treatments

**Figure 2:**
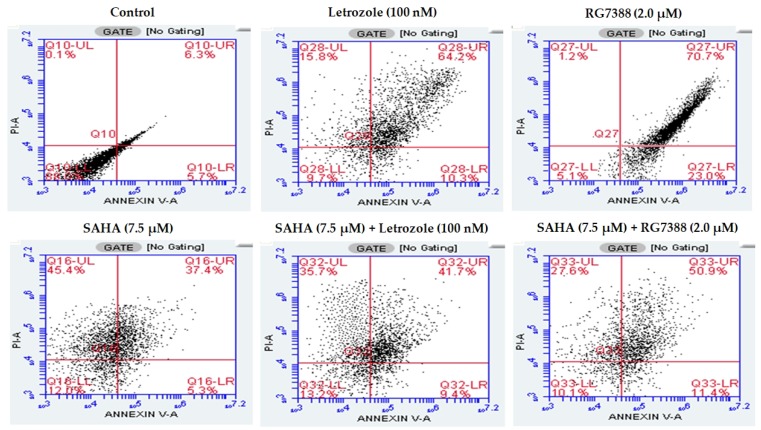
Apoptosis, necrosis, and necroptosis analysis by flow cytometry using Annexin V/PI double staining. MCF-7 cells were treated with SAHA (7.5 μM), letrozole (100 nM), and RG7388 (2.0 μM) individually and in combinations (SAHA + letrozole and SAHA + RG7388) for 24 h.

In addition to the TBDE method, we used flow cytometry analysis to assess cell death induced by individual and combination treatments with SAHA, letrozole, and RG7388. Flow cytometry analysis using Annexin V/Propidium Iodide staining 24 h post-treatment, was used to determine the percentages of apoptotic, necroptotic, and necrotic cell (Figure 2). Cells negative for both PI and Annexin V staining were viable cells (in the lower left quadrant; Q3); PI-negative Annexin V-positive cells were early apoptotic cells (in the lower right quadrant; Q4); PI-positive Annexin V-positive cells were primarily late apoptosis/necrotic cells (in the upper right quadrant; Q2); and the PI-positive but Annexin V-negative cells were necrotic cells (in the upper left quadrant; Q1). As anticipated, RG7388 was able to produce apoptotic cell death showing 70.7% of cells in Q2, and similarly letrozole treatment was also showing apoptosis mediated cell death with nearly 64.2% of cells in Q2. However, the total amount of cells found in the Q1 was significantly higher (45%) in SAHA (7.5 µM) compared to other treatments. Detection of greater amounts of PI stained dead cells in the SAHA treatment suggested that the cell death mechanisms triggered by SAHA may be different compared to RG7388 treatments. In order to discriminate the mechanisms triggered by SAHA, letrozole, and RG7388 treatments, a fluorescence microscope imaging analysis and fluorometric assays using SYTOX green and DEVD-*amc* were performed, and the results are presented in the next section.

### 3.3. Effect of SAHA on the Induction of Necroptosis

**Figure 3:**
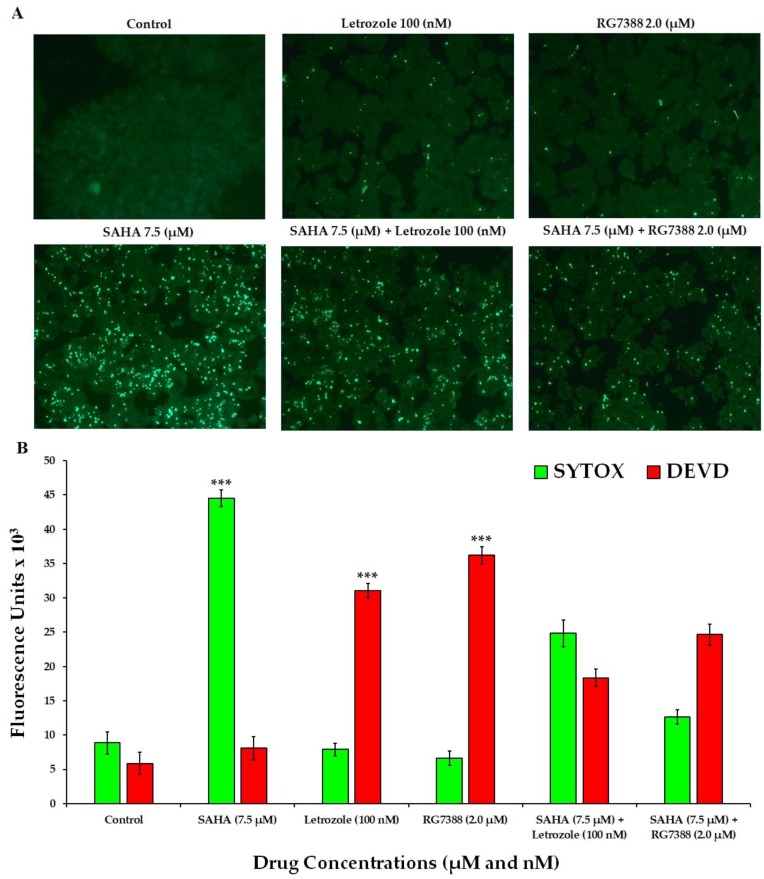
Analysis of necroptosis versus apoptosis in MCF-7 cells. (**A**) Confirmation of necroptosis versus apoptosis in MCF-7 cells treated with SAHA (7.5 μM), letrozole (100 nM), and RG7388 (2.0 μM) individually and in combinations (SAHA + letrozole and SAHA + RG7388) using the SYTOX green staining method. (**B**) Analysis of necroptosis and apoptosis in MCF-7 cells with SAHA (7.5 μM), letrozole (100 nM), and RG7388 (2.0 μM) individually and in combinations (SAHA + letrozole and SAHA + RG7388) for 24 h, with SYTOX green and DEVD-*amc* using a spectrofluorometric assay in a 96-well plate. Data are presented in the bar graph are mean ± SD, from minimum of three independent experiments. The level of significance is indicated as *** *p* < 0.001 compared to control.

To determine the actual mechanism of cell death induced by SAHA, a fluorescence imaging analysis was conducted using the SYTOX green staining technique. SYTOX Green staining is a method that allows for the determination of caspase-independent cell death/necrosis, as it stains accessible DNA when membrane integrity is compromised. Experiments with SYTOX green clearly revealed that treatment of MCF-7 cells with SAHA was inducing Necroptosis (Figure 3A). As a result, cells treated with a 7.5 µM concentration of SAHA showed significant levels of death, since these cells produced high levels of green fluorescence from SYTOX green. On the other hand, RG7388 induced very little green fluorescence with SYTOX green. For further verification, the fluorescence intensities were measured using the 96-well plate assay, with the inclusion of DEVD-*amc* substrate for the assessment of apoptosis through caspase-3/7 activation. 

The level of fluorescence from SYTOX green in 7.5 µM SAHA-treated MCF-7 cells was 44,489 ± 1233 units compared to 7917 ± 920 and 6621 ± 1049 units of fluorescence in letrozole and RG7388 treated cells, respectively (Figure 3B). On the other hand, the green fluorescence from DEVD-*amc* in RG7388 treated cells was 36,176 ± 1224 units. However, the SAHA-treated cells showed low fluorescence in the range of 8077 ± 1694 units only with DEVD-*amc* experiments. Letrozole treatment showed 31,066 ± 1033 units of fluorescence with DEVD-*amc,* while the fluorescence from SYTOX was around 7917 ± 920. In the combination treatment (SAHA + RG7388), SYTOX was able to show fluorescence due to the caspase-independent cell death caused by SAHA, in addition to the caspase-dependent effects of RG7388. These results indicated that the types of cell death induced by SAHA and RG7388 are different. Hence it was very interesting to further explore the intracellular mechanisms because, SAHA induced p21 mediated cell cycle arrest using p53 independent pathways, which might be leading to induction of necroptosis, while RG7388 was able to induce apoptosis without elevation of p21. 

### 3.4. Effect of SAHA and RG7388 on the Migration of MCF-7 Cells

**Figure 4:**
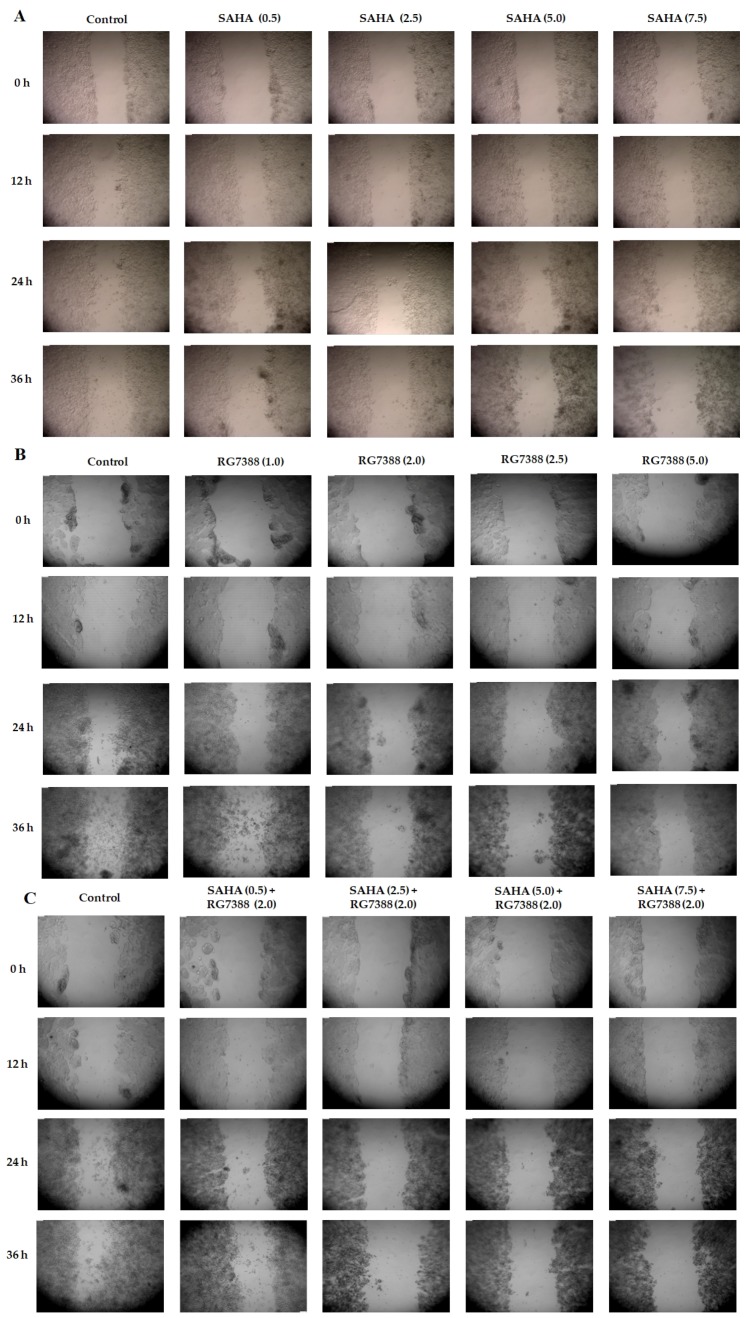
Inhibitory effects of SAHA and RG7388, in individual or combinations treatments, on MCF-7 cell migration during scratch assays. Representative images of MCF-7 cells captured at 0, 12, 24, and 36 h of treatment with different drugs. (**A**) Cells treated with 0.5, 2.5, 5.0, and 7.5 µM of SAHA. (**B**) Cells treated with 1.0, 2.0, 2.5, and 5.0 µM of RG7388. (**C**) Cells treated with SAHA and RG7388 (2.0 µM) in combinations.

Using the scratch assay, we investigated the effects of SAHA, RG7388, and their combination on cell migration in a dose dependent manner from 0–36 h of incubation. The results shown in Figure 4A–C, indicate that there were significant differences in cell migration abilities. The treatments of MCF-7 cells with SAHA and RG7388, individually or in combination, were able to significantly reduce the migration abilities of cancer cells, in addition to inducing the cell death.

### 3.5. Effect of SAHA on the Expression of Cell Cycle Related Genes Using MCF-7 Cells

**Figure 5:**
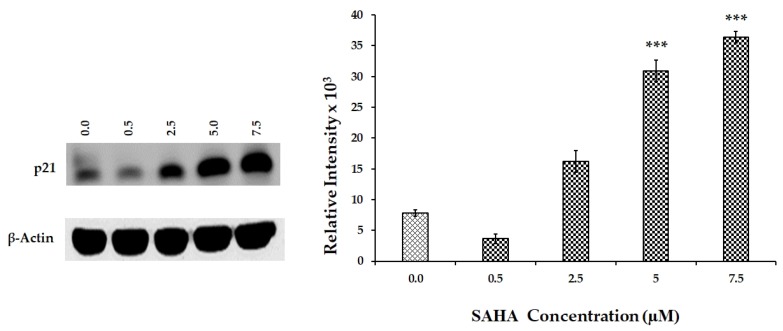
Dose dependent response of p21 expression in SAHA (0.5–7.5 µM) treated MCF-7 cells. The level of significance is indicated as *** *p* < 0.001 compared to control.

**Figure 6:**
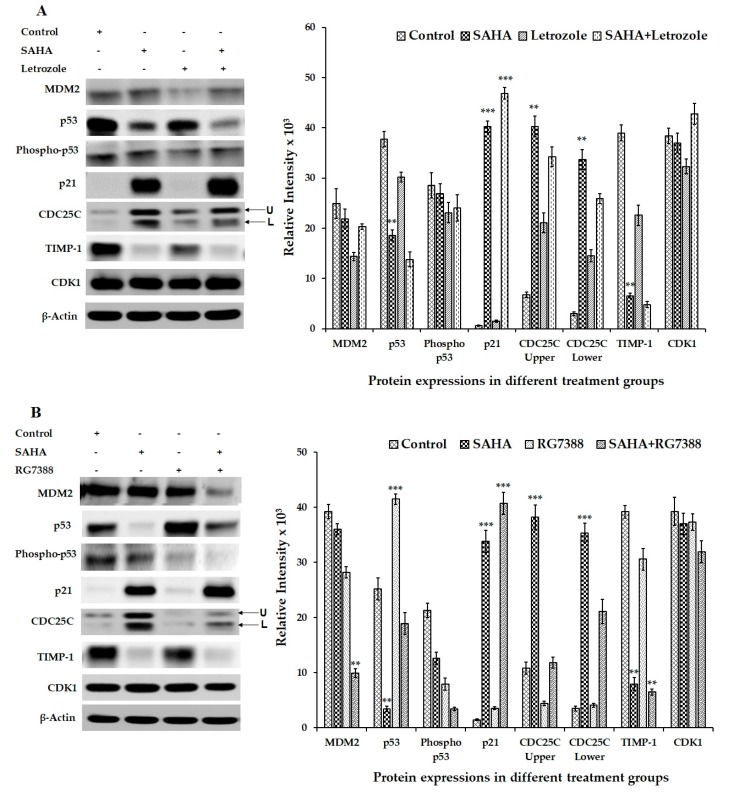
Representative western blot images showing the changes in the levels of cell cycle-related proteins in MCF-7 cells after SAHA, letrozole RG7388 and combination treatments. (**A**) Modulation in the levels of cell cycle-related gene proteins such as MDM2, p53, phospho-p53, p21, CDC25C, TIMP-1, and CDK1 in MCF-7 cells treated with SAHA (7.5 μM), letrozole (100 nM) and SAHA + letrozole combination for 24 h. (**B**) Modulation of cell-cycle related protein expressions in MCF-7 cells after SAHA (7.5 μM), RG7388 (2.0 μM) and SAHA + RG7388 treatment for 24 h. The right panel represents the band intensity of the cell cycle proteins normalized to that of β-actin by ImageJ software. Data are presented as means ± SD from a minimum of three independent experiments. The level of significance is indicated as ** *p* < 0.01 and *** *p* < 0.001 compared to control.

Western blot experiments were conducted to detect the changes in the levels of cell cycle-related proteins such as MDM2, p53, phospho-p53, p21, CDC25C, TIMP-1, and CDK1 after SAHA treatment. Initially, the levels of p21 were analyzed after treating the cells with different concentrations of SAHA, which provided a dose dependent elevation of p21. Western blot analysis revealed gradual increase in p21 levels from 2.5–7.5 µM concentrations of SAHA treatment compared to the control cells (Figure 5). On the other hand, when MCF-7 cells were treated with letrozole or RG7388 only basal level expression of p21 was observed (Figure 6A,B). As anticipated, the levels of p53 were significantly elevated after RG7388 (2.0 μM) treatment of MCF-7 cells for 24 h. However, RG7388 treatment was not able to elevate p21 expression following inhibition of MDM2, though there was clear evidence of total p53 elevation, but the levels of phospho-p53 were significantly lower compared to controls (Figure 6B). Letrozole treatment did not produce any significant alteration in the levels of p53 (Figure 6A). Interestingly, the levels of CDC25C (Cell Division Cycle 25C Phosphatase), that is known to trigger entry of cells into mitosis in the cell cycle by dephosphorylating cyclin B-Cdk1, were elevated significantly during SAHA treatment (Figure 6A,B). However, RG7388 treatment showed no changes in the levels of CDC25C (Figure 6B). CDK levels were unaltered following SAHA, letrozole, RG7388, or combination treatments. Furthermore, TIMP-1 levels were significantly lowered in SAHA treatment (Figure 6A) compared to control and RG7338, which was not able to produce significant alteration in the TIMP-1 levels.

### 3.6. Effect of SAHA on the Expression of Apoptosis-Related Genes Using MCF-7 Cells

**Figure 7:**
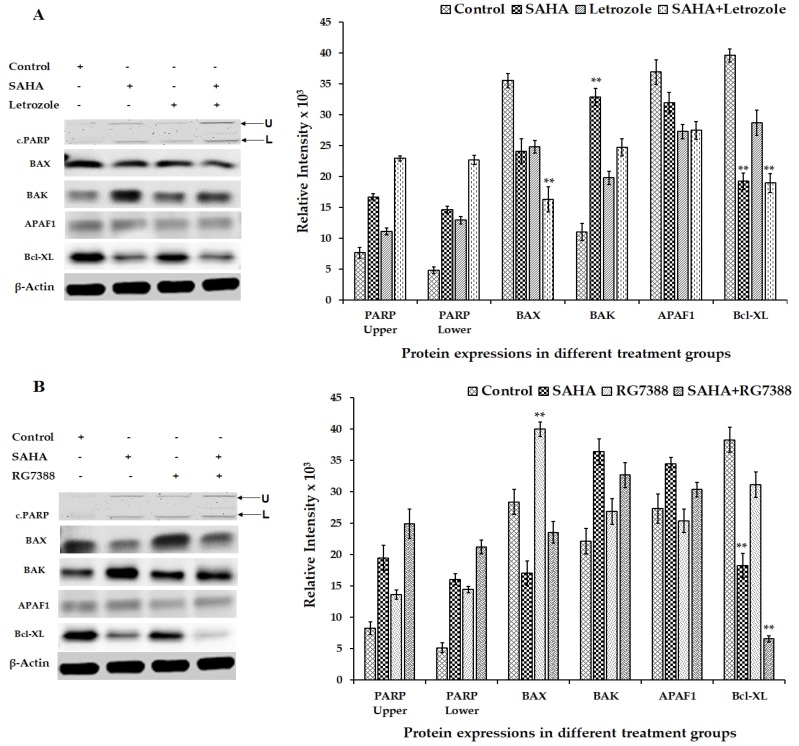
Representative western blot images showing the changes in the levels of apoptosis-related proteins in MCF-7 cells after SAHA, letrozole, RG7388 and combination treatments. (**A**) Modulation in the levels of apoptosis-related gene proteins such as cleaved PARP, BAX, BAK, APAF1, and Bcl-XL in MCF-7 cells treated with SAHA (7.5 μM), letrozole (100 nM) and SAHA + letrozole for 24 h. (**B**) Modulation of apoptosis-related protein expressions in MCF-7 cells after SAHA (7.5 μM), RG7388 (2.0 μM) and SAHA + RG7388 treatment for 24 h. The right panel represents the band intensity of apoptosis proteins normalized to that of β-actin by ImageJ software. Data are presented as means ± SD from a minimum of three independent experiments. The level of significance is indicated as ** *p* < 0.01 compared to control.

In addition to the changes noted in cell cycle regulatory proteins, apoptosis-related proteins showed significant changes (Figure 7A,B). For example, the levels of BAX were significantly elevated in RG7388 treated cells, whereas there was no significant change in the levels of BAX in SAHA and letrozole treated cells (Figure 7A). Unexpectedly, the level of BAK was noticeably elevated in SAHA treatment, whereas RG7388 treatment did not show any significant alterations compared to the control (Figure 7A,B). Interestingly, APAF1 levels did not change subsequent to treatment with SAHA, letrozole, and RG7388 (Figure 7A,B). Though there appears to be cleavage of PARP following RG7388 and combination treatments, the low levels of PARP that were detectable in the MCF-7 cells (Figure 7A,B) made it difficult to draw any meaningful conclusions. Another interesting result from our experiment was the significant decrease in the levels of Bcl-XL, while a noticeable elevation of BAK was observed after 24 h, though BAX levels were unaltered by the SAHA treatment (Figure 7A,B).

### 3.7. Effect of Drug Treatments on Necroptosis-Related Gene Expressions Using MCF-7 Cells

**Figure 8:**
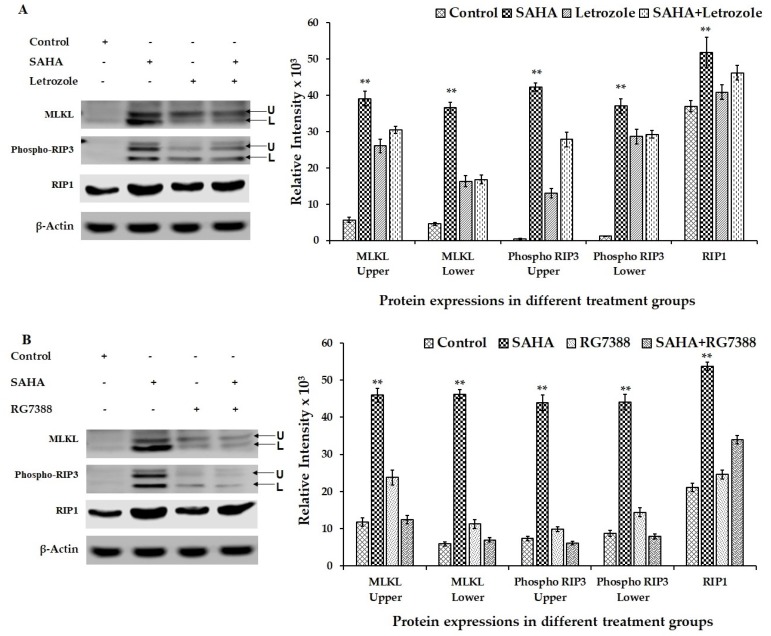
Representative western blot images showing the changes in the levels of necroptosis-related proteins in MCF-7 cells after SAHA, letrozole RG7388 and combination treatments. (**A**) Modulation in the levels of necroptosis-related gene proteins such as MLKL, phospho-RIP3, and RIP1 in MCF-7 cells treated with SAHA (7.5 μM), letrozole (100 nM) and SAHA + letrozole for 24 h. (**B**) Modulation of necroptosis-related protein expressions in MCF-7 cells after SAHA (7.5 μM), RG7388 (2.0 μM) and SAHA + RG7388 treatment for 24 h. The right panel represents the band intensity of apoptosis proteins normalized to that of β-actin by ImageJ software. Data are presented as means ± SD from minimum of three independent experiments. The level of significance is indicated as ** *p* < 0.01 compared to control.

**Figure 9:**
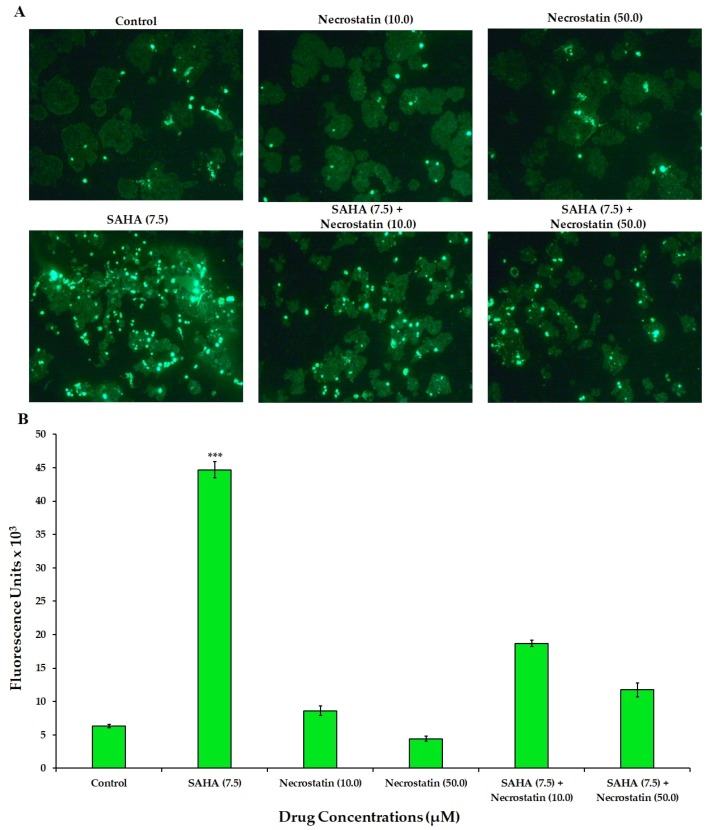
Analysis of necroptosis in MCF-7 cells. (**A**) Confirmation of necroptosis in MCF-7 cells treated with SAHA (7.5 μM), Necrostatin (10, 50 μM) individually, and in combinations SAHA + Necrostatin (10 μM) and SAHA + Necrostatin (50 μM) using the SYTOX green staining method. (**B**) Confirmation of necroptosis in MCF-7 cells treated with SAHA (7.5 μM), Necrostatin (10, 50 μM) individually, and in combinations SAHA + Necrostatin (10 μM) and SAHA + Necrostatin (50 μM) with the SYTOX green staining method using a spectrofluorometric assay in a 96-well plate. The level of significance is indicated as *** *p* < 0.001 compared to control.

**Figure 10:**
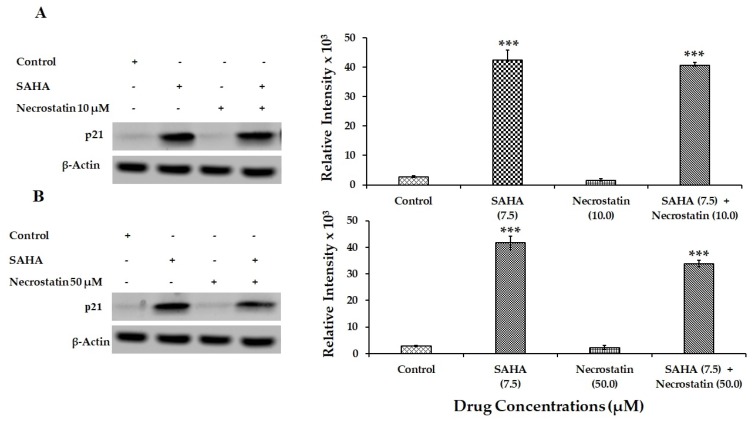
Effect of SAHA and Necrostatin treatments on p21 expression in MCF-7 cells. (**A**) Effect of SAHA (7.5 µM) and Necrostatin (10 µM) on p21 levels in MCF-7 cells after 24 h treatment. (**B**) Modulation of p21 protein levels in MCF-7 cells after 24 h treatments with SAHA (7.5 µM) Necrostatin (50 µM) and combination. The level of significance is indicated as *** *p* < 0.001 compared to control.

RCD induced by SAHA treatment exhibited ample evidence to suggest that MCF-7 cells may go through necroptosis after 24 h of treatment with HDACi rather than going through other types of cell death including apoptosis. Therefore, MLKL, phospho-RIP3 (phospho-Receptor Interacting Protein Kinase 3), and RIP1 (Receptor Interacting Protein 1) were analyzed as key cellular mediators, and therefore as markers of necroptosis. Evidently, phospho-RIP3 and MLKL levels were significantly elevated compared to untreated control cells (Figure 8A,B). In addition to the elevation in phospho-RIP3 levels, RIP1 levels were also found to be significantly elevated in SAHA-treated cells. However, letrozole and RG7388 treatments were able to show only slight elevation in the levels of the RIP1. When cells were incubated with SAHA in the presence of necrostatin (50 μM) for 24 h, the level of cell death shown by the elevation of SYTOX fluorescence was significantly reduced (Figure 9A), as it was also confirmed using the 96-well plate assay (Figure 9B). Interestingly, when the MCF-7 cells were pre-incubated for 15 min with necrostatin (50 μM), before treatment with SAHA (7.5 μM), the elevation of p21 caused by the HDACi was able to significantly reduce the elevation of p21 (Figure 10). Necrostatin alone did not produce any noticeable effects on the p21 expression.

## 4. Discussion

Our results obtained from MCF-7 cells, following SAHA treatments, revealed interesting mechanisms for HDACi-induced cell death. Several reports in the literature have indicated that, SAHA treatment was able to induce a significant amount of cell death following 24 h treatments, and it was suspected to occur mainly through inhibition of cell proliferation and consequently trigger the cell death process caused by the elevation of p21 levels in various cancer cell lines. In our experiments, SAHA was also able to induce significant elevation in p21 expression as well as cell death in a concentration- and time-dependent manner. Very similar to the results presented in this study, several other investigators have suggested that the inhibition of cell cycle progression by SAHA occurs primarily through induction of p21 (WAF1/CIP1) protein expression that was caused by histone hyper acetylation [36]. In this regard, it is very well known that several HDAC inhibitors have revealed significant anti-proliferative activities against both leukemic and solid tumor cell lines [1]. Previous reports have also shown that, cancer cells treated with SAHA undergo a dose-dependent cell cycle arrest in G1 phase at lower concentrations, and at higher concentrations further arrest was observed in the G2-M phase of the cell cycle. So far, several mechanisms have been proposed for the induction of cancer cell death by SAHA, including ROS production, stimulation of cytochrome c-mediated apoptosis, and autophagy [37,38]. In all these earlier studies the cell cycle arrest, apoptosis, and necrosis were reported to clearly associate with the up-regulation of p21 (WAF1/CIP1) via p53-independent mechanisms. 

During our experiments, though the TBDE assay and the flow cytometry results confirmed significant level of cell death of MCF-7 cells after SAHA treatment, the exact nature of this cell death was not evident. Our experiments with SYTOX green and DEVD-*amc* green fluorogenic substrates, have suggested that the induction of cell death by SAHA is due to necroptosis, which is a caspase-independent form of RCD. The fluorescence signal obtained from SYTOX green was maximum, due to the dye penetrating the plasma membrane and traversing the cytoplasm of the cells and binding to the nucleic acid. On the other hand, the cleavage of DEVD-*amc* by caspase 3/7, which typically occurs during apoptosis was minimal after the treatment of cells with 7.5 µM SAHA. However, the MDM2 inhibitor RG7388, which can produce p53-dependent induction of p21 led to PARP cleavage and completion of apoptotic cell death. Thus, it was interesting to note that two different drugs, which are known to induce p21 expression; SAHA through p53 independent mechanism and RG7388 through p53 dependent mechanism, both produced cell death through two distinct mechanisms. Typically, RG7388 relieves p53 from MDM2 binding, which eventually leads to the transcriptional transactivation of p21 gene by p53 and consequent cell cycle arrest or apoptosis. Certainly, the inability for RG7388 cells to induce p21 in MCF-7 cells was not expected, however, induction of apoptosis by RG7388 is a very interesting new finding and that appears to be through the inhibition of MDM2. This may be partially due to a mechanism that is unknown at this time, which leads to the elevation of BAX after MDM2 blockade. In contrast to the mechanism mediated by RG7388, the induction of p21 by HDAC inhibitors is shown to be through direct activation of the p21 promoter, due to histone hyper acetylation and consequent expression of the p21 gene [39], that might be independent of caspases and BAX. During SAHA-induced cell death, TIMP-1 may be involved since it’s levels were significantly lowered. TIMP-1 has been shown to protect tumor cells from chemotherapy-induced RCD both in the in vitro and in clinical experiments. As a result, TIMP-1 was proposed as a potential marker for prediction of response to chemotherapy in breast cancer [40]. In addition, some of the earlier studies have concluded that the p53 independent mechanisms of cell death in cancer cells have been mediated through BAK, which was markedly elevated in SAHA-treated MCF-7 cells [41]. Therefore, closer analysis of TIMP-1 and BAK expression, and their modulation during SAHA and RG7388 treatments in MCF-7 cells may offer additional mechanistic insights related to the cell death events caused by these drugs. 

The molecular effects of SAHA observed in MCF-7 cells could be due to one of the p21-dependent mechanisms that resulted in the inhibition of cell cycle progression, and cell proliferation leading to necroptosis in a caspase-dependent manner. So far, caspase-dependent apoptosis is considered as the primary route to programmed cell death (PCD) in most physiological settings. In the recent years, research studies concerning caspase-independent, non-apoptotic forms of PCD have been advancing, which have enabled the identification of backup mechanisms to allow the cells to undergo suicide or RCD under conditions where the caspase machinery is defective or inhibited [33]. Thus, necroptosis that is mediated by the RIP1 and RIP3 kinases has emerged as an important and physiologically relevant response mechanism to trigger cell death following various drug treatments. Interestingly, our experiments revealed elevation of RIP1, phospho-RIP3 and MLKL levels in MCF-7 cells following SAHA treatment. RIP1 activation and phospho-RIP3 elevation have been shown as critical triggers in the induction of necroptosis, particularly in circumstances where the caspase cascade is defective. Moreover, depending on the circumstances, sometimes the role of RIP1 could be non-essential also for causing RCD. For example, RIP1 deletion may even promote necroptosis under certain conditions, with the activation of RIP3 alone [42,43]. However, Tumor necrosis factor-alpha (TNF-α)-induced necroptosis requires the transduction of signals from RIP1 to RIP3, which permits RIP3 to recruit more phospho-RIP3 to form homo-oligomerization/aggregation and auto-phosphorylation of RIP3. The Phospho-RIP3 in the oligomeric (complex) is known to further recruit and phosphorylate a mixed lineage kinase domain-like (MLKL) pseudokinase to make a phospho-RIP3-MLKL complex, which is also called necrosome or ripoptosome [43]. During the regulation of necroptosis, reactive oxygen species (ROS) also participate to enhance necrosome formation in many but not in all types of cancer cells that undergo necroptosis [44]. As described in the introduction section, activation of proteases such as HtrA2/Omi and UCH-L1, and consequent proteolysis are also found to be essential for necroptosis [33]. Similarly, necroptosis appears to be independent of PARP and executed without it. Thus, in our MCF-7 experimental system, the phospho-RIP3 and MLKL elevation detected following SAHA treatment appear to be driving the necroptosis events leading to ultimate cell death. On the contrary, inhibition of MDM2 by RG7388 appears to be clearly inducing apoptosis, without significant elevation of p21 in MCF-7 cells. Though, letrozole and RG7388 treatments showed only marginal elevation of phospho-RIP3 levels, due to the noticeable elevation of BAX following treatment with RG7388, it is suspected that cell death caused by MDM2 inhibition could be due to mechanisms other than the necroptosis and more likely due to apoptosis.

Generally, drugs that target necrosis or necroptosis can be more efficient in treating breast cancer cells that are resistant to apoptosis. Our current data suggest that SAHA treatment causes upregulation of p21 protein expression via p53-independent pathway, and subsequently necroptosis through phospho-RIP3-dependent pathway. MCF-7 cells do not show strong evidence of apoptosis following SAHA treatment, as evidenced by the lack of a fluorescence signal obtained with the DEVD-*amc* substrate, and also due to the lack of BAX elevation, which are consistent with some previous reports that can be found in the literature. Since SAHA treated cells showed a strong increase in protein expression of p21 and inhibition of CDK, this may also be involved in the induction of necroptosis. Several chemotherapeutic drugs, in particular DNA damaging agents, have been shown to induce necroptosis in cancer cells [45]. This type of alternative mechanism is important because, apoptosis could be defective in many cancer cells due to p53 mutations or other genetic and epigenetic alterations that can influence key apoptotic regulators [46]. For example, etoposide, a topoisomerase inhibitor was shown to suppress cIAP expression, thereby inducing necroptosis through a RIP1-containing complex [47]. The anti-mitotic drug taxol was also shown to induce RIP1-dependent necroptosis that was mediated through dual phosphorylation of Fas-associated protein with death domain (FADD) by Aurora-A and Plk1 [48]. Other chemotherapeutic agents such as 5-Fluorouracil (5-FU), etoposide, and camptothecin were shown to induce RIP1/MLKL-dependent, but RIP3-independent necroptosis in caspase-3-deficient colorectal cancer cells [49]. On the other hand, cisplatin caused RIP3-dependent necroptosis in apoptosis-resistant esophageal cancer cells through necrosome formation that was triggered by autocrine TNF-α signaling [50]. Because of its significant role, epigenetic silencing of RIP3 was reported to contribute to the resistance for 5-FU, cisplatin, camptothecin, and etoposide in colon and breast cancer cells. Furthermore, necroptosis was also shown to be induced by kinase inhibitors, natural products, or immune modulators. For example, Sorafenib, which is a FDA-approved multi-kinase inhibitor drug, was shown to produce RIP1-dependent necroptosis in prostate cancer cells [51]. A combination of Sorafenib with the Givinostat, which is a HDACi, induced RIP1-dependent necroptosis through ROS production and activation of the BH3-only containing Bcl-2 family protein called Bim [52]. Another pan-kinase inhibitor, staurosporine, a widely used apoptosis inducer, was found to promote RIP3/MLKL-dependent necroptosis in U-937 lymphoma cells under caspase-compromised conditions [53]. Interestingly, necroptosis induced by these agents was often found to be accompanied by autophagy, which is also believed to be responsible for the suppression of apoptosis and rendering cells towards necroptosis. Thus, many of the existing reports in the literature have suggested that, the status of RIP1 and RIP3 can determine the level and extent of necroptosis in cancer cells [54]. In many cancer cell lines SAHA initiates cell death by activation of mitochondria-mediated death pathways that are characterized by cytochrome c release and formation of reactive oxygen species (ROS), without the activation of key caspases such as caspase-8 or caspase-3. Earlier studies have provided evidence that mitochondrial disruption is achieved by means of cleavage of the BH3-only pro-apoptotic Bcl-2 family member Bid [55], which is another key mediator of SAHA-induced mitochondrial membrane disruption. Bid cleavage was not blocked by caspase inhibitors or overexpression of Bcl-2, but depended on the transcriptional regulatory function of SAHA. These findings indicate that drug-induced Bid cleavage can occur upstream of mitochondrial perturbation, and that Bid cleavage might be an important process in SAHA-mediated cell death [55]. 

**Figure 11:**
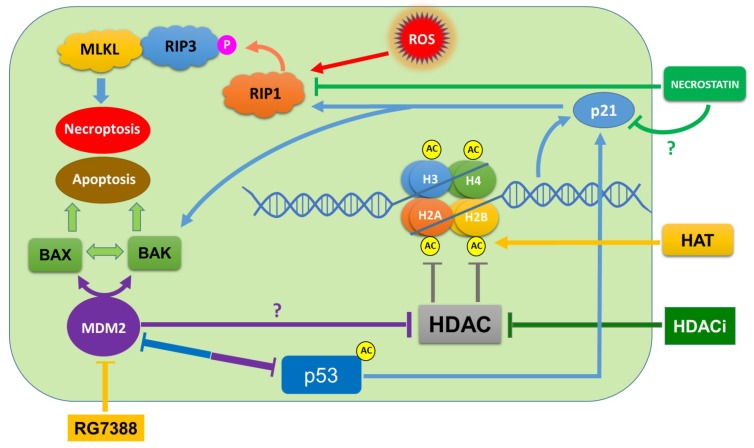
Various pathways that might be involved in SAHA- and RG7388-induced cell death. SAHA-induced necroptosis is mediated through phospho-RIP3, and RG7388-mediated apoptosis is possibly through p53- and p21-independent mechanisms but mediated through BAX and BAK.

In conclusion, our comparative drug treatment results indicated that SAHA and letrozole during combination treatments did not produce any greater cell death than the individual treatments, since apoptosis markers such as BAX were not elevated, and PARP was not cleaved significantly by letrozole treatment. The novel finding of our study is that, SAHA strongly induces necroptosis through elevation of p21, phospho-RIP3, and MLKL levels in MCF-7 cells. We can also see from our results that apoptosis markers such as BAX were not elevated, and PARP was not cleaved by SAHA in 24 h. The analysis of SYTOX green binding to the nuclear component along with phospho-RIP3 elevation provides the evidence for the induction of necroptosis by SAHA in MCF-7 cells. SAHA-induced upregulation of p21 (WAF1/CIP1) protein expression is believed to be due to the histone hyper acetylation [56,57], and induction of genes which are involved in regulation of cell cycle arrest and necroptosis in MCF-7 cells. Thus, our experiments so far have permitted us to partially elucidate the SAHA-induced inhibition of cell proliferation and necroptosis of MCF-7 cells. From our experimental results, it is evident that different mechanisms are responsible for SAHA and RG7388 mediated cell death of MCF-7 cells, as outlined in Figure 11, which may cross-talk during the execution process. Developing a clear understanding of the differences in the intracellular mechanisms is important while inducing cell cycle arrest or RCD, because certain gene defects such as MDM2 overexpression, p53 status, or epigenetic alterations may determine the actual pathway that is triggered for causing cell death. Additional studies related to pathways linking p21 to RIP1 and RIP3, leading to necroptosome formation is necessary to fully conclude the mechanism of SAHA-induced cell death.
